# Distinct patterns of fasting plasma glucose and lipid profile levels over time in adults tested positive for HIV on HAART in Shanghai, China, revealed using growth mixture models

**DOI:** 10.3389/fmed.2022.1071431

**Published:** 2023-01-17

**Authors:** Jingjing Lang, Xin Xin, Panpan Chen, Zhen Ning, Shaotan Xiao

**Affiliations:** ^1^School of Public Health, Fudan University, Shanghai, China; ^2^Pudong New Area Center for Disease Control and Prevention, Shanghai, China; ^3^Pudong Institute of Preventive Medicine, Fudan University, Shanghai, China; ^4^Shanghai Center for Disease Control and Prevention, Shanghai, China

**Keywords:** HIV, HAART, growth mixture model, fasting plasma glucose, lipid

## Abstract

**Objectives:**

This study sought to identify potential change patterns and predictors of fasting plasma glucose (FPG) and lipid levels after initiating highly active antiretroviral therapy (HAART).

**Methods:**

A retrospective cohort study was conducted on 1,572 patients tested positive for HIV who initiated HAART between January 2010 and October 2020 in Shanghai, China. The growth mixture models (GMM) were used for capturing subgroups of FPG trajectories as well as triglyceride (TG) and total cholesterol (TC) dual-trajectories. Multinomial logistic regression models identified correlates of given trajectories.

**Results:**

The median follow-up time was 2.0 years (IQR 1.0–4.7). Three FPG trajectory subgroups were identified as FPG low-stable (62.3%), medium-stable (30.5%), and high-increasing (7.2%). Furthermore, three subgroups of TG and TC dual-trajectories were identified as TG and TC high-slight increasing (13.7%), low-rapid increasing (27.6%), and a subgroup of medium-stable TC and slight-decreasing TG (58.7%). Older age, high TG, FPG, BMI, CD4 count of <200 at baseline, and initial use of zidovudine (AZT) and protease inhibitors (PIs) helped to identify the class with increasing glucose or lipid metabolism trajectories.

**Conclusion:**

The change patterns of plasma glucose and lipid in patients tested positive for HIV were heterogeneous and tailored interventions should be considered in specific subgroups.

## 1. Introduction

Since the introduction of antiretroviral therapy (ART), survival rates for patients tested positive for HIV have improved, and life expectancy has increased significantly. AIDS-related mortality rates have fallen, whereas deaths due to non-AIDS-defining diseases such as cardiovascular diseases (CVDs) have risen (1). ART and later highly active antiretroviral therapy (HAART) have some adverse effects on glucose and lipid metabolism (2), resulting in an increased risk of CVD (3).

Currently, there is no consensus on the association among HIV infection, ART, and the development of diabetes mellitus (DM) (2). In several studies (4, 5), the association of HIV infection and ART with an increased risk of DM has been suggested with potential mechanisms including chronic inflammation and ART-induced mitochondrial dysfunction (6, 7). In contrast, others reached different conclusions (8). A meta-analysis showed that in studies with a mean duration of ART of ≥18 months, ART was associated with a significant increase in fasting plasma glucose (FPG) levels but not in studies with a mean duration of ART of <18 months (9), suggesting that the impact of antiretroviral medications on blood glucose might take time to manifest. In addition, the effects of specific ART classes and antiretrovirals on DM development have not been thoroughly understood (2, 10).

Similarly, although the association between ART and hyperlipidemia has been well-evidenced, there was no coherent conclusion on the long-term changing patterns of blood lipids after initiating HAART. It is generally accepted that the lipid phenotype is characterized by decreasing the levels of high-density lipoprotein cholesterol (HDL) and increasing the levels of triglycerides (TG) and low-density lipoprotein cholesterol (LDL). However, no consistent results have been obtained for the changing pattern of total cholesterol (TC), shown to be elevated in some studies but decreased in others (11, 12). Furthermore, though newer drugs of ART were suggested to have more favorable effects on lipid metabolism than older ones, such as efavirenz (EFV) and protease inhibitors (PIs) (13, 14), recent studies have reported that using newer drugs, such as tenofovir alafenamide (TAF) and integrase strand transfer inhibitors (INSTIs), might lead to more significant increases in TG, TC, and HDL (15, 16).

Focusing on potential patterns of longitudinal blood glucose and lipid changes might help provide a more nuanced picture of metabolic changes. Studies in the general population have proved that latent change patterns, such as inverse U-shaped trajectory and high growth rate of plasma glucose and lipid levels, played an essential role in the development of CVD (17–19). However, to our knowledge, there is a lack of research characterizing the potential trajectories of plasma glucose and lipid levels in patients tested positive for HIV on HAART, which would help to further uncover the association between HAART regimens and changes in glucose and lipid metabolism. Several statistical methods have been developed for identifying unobserved subpopulations of trajectories of quantitative variables over time (20). One of them is the growth mixture model (GMM), which is a method for capturing heterogeneity in individual trajectories and identifying multiple unobserved subgroups of participants with similar trajectories (21). This study aimed to identify latent subgroups of plasma glucose and lipid levels trajectories using GMM and evaluate their associations with both traditional risk factors and HIV-specific factors, especially HAART regimens.

## 2. Materials and methods

### 2.1. Data sources

Data in this retrospective cohort study were drawn from two national web-based databases in China, namely, the HIV/AIDS Comprehensive Response Information Management System (CRIMS) and the HIV/AIDS case reporting system (CIS), as documented elsewhere (22). Demographic characteristics of patients (e.g., age, gender, height, weight, and HIV transmission route) and disease-related data (e.g., TC, TG, and FPG measurements, CD4 cell count, HIV viral load, and ART regimens) were collected for this study.

Fasting blood samples were extracted and analyzed. FPG and lipids were measured by a fully automatic biochemistry analyzer (Roche, Basel, Switzerland) using an enzymatic method. Absolute CD4+T lymphocytes were counted by a FACS Calibur flow cytometer (Becton-Dickinson, USA) within 24 h after blood sample processing. Plasma HIV-RNA viral load was measured using an automated Abbott real-time HIV-1 assay (Abbott, USA).

### 2.2. Eligible participants

Data were collected from January 2010 to August 2021. The eligibility criteria were patients with HIV newly diagnosed between January 2010 and October 2020, age older than 18 years, ART-naive, non-pregnant, and newly initiated on a HAART regimen consisting of a pair of nucleoside reverse transcriptase inhibitors (NRTIs) as backbone drugs together with non-nucleoside reverse transcriptase inhibitors (NNRTIs), PIs, and INSTIs as core drugs. We excluded patients with missing height, weight, TC, TG, FPG, and CD4 count at the baseline and those with less than two follow-up visits in different years.

### 2.3. Definitions

Body mass index (BMI, kg/m^2^) was categorized as underweight (<18.5), normal (18.5 to <24), and overweight or obese (≥24). High FPG, TC, and TG were defined as FPG of ≥6.1 mmol/l, TC of ≥5.2 mmol/l, and TG of ≥1.7 mmol/l, respectively, according to the NCEP ATP III criteria.

### 2.4. Statistical analysis

For statistical descriptions, continuous variables were expressed as means and standard deviations if normally distributed, or medians and interquartile ranges (IQR) if not. Categorical variables were expressed as numbers and proportions.

FPG, TG, and TC measurements at visits occurring between 1 month and 7 years after HAART initiation were used to calculate subsequent observations. Multiple measurements available for a single year were averaged, and then log-transformed because of their skewed distribution. Using Mplus 8.3 (23), FPG trajectories over time were modeled by the GMM method, and distinct subgroups that followed similar patterns were identified. To capture the interaction of TG and TC development, parallel process GMMs were conducted to determine the shared developmental trajectories of both lipid levels.

The analytical procedure was planned following frameworks suggested in previous studies (24, 25). First, different growth curve models (GCMs) (i.e., linear, quadratic, and latent basis models) were fitted to select the best-fit model for the following steps (26). Then increasingly free models were conducted following the path of going from the most constrained group-based trajectory models (GBTM) without random effects to more flexible latent class growth analysis (LCGA) models with different residual variance error structures and GMM with varying covariance structures of the random effects by gradually reducing the model restrictions (25). We used a class-specific unstructured covariance matrix to avoid any assumptions about the covariance structure (27). Moreover, a greater range of start values was specified to avoid local solutions in the estimation of GMMs (28).

Model fit criteria curve behaviors were identified to determine whether to fit more complex models, a plateauing behavior of the fit statistics indicating possible covariance misspecification (29). Seminal studies in this field proposed several criteria that were used to select the best latent class number K (25). Adjusted Lo-Mendell–Rubin likelihood ratio tests (aLMR) and Vuong-Lo-Mendell-Rubin tests (VLMR) were taken for comparison between class K models and class K-1 models. A significant *p*-value (*p* < 0.05) represented the superiority of the class K model over the class K-1 model. Bayesian information criterion (BIC) was also evaluated when comparing different models: a small number of BIC indicated a better-fitting model. Then, the average posterior probability of assignment (APPA) close to 1 (ideally >0.7), a sufficient sample size (>5% of the sample) for each trajectory group, and a large value of entropy was indicative of good model parsimony and adequacy. Finally, trajectory groups were marked by the relative baseline level (low, medium, and high) and change tendency (increasing, decreasing, and stable).

Baseline information was compared between subgroups using either the chi-square test or Fisher's exact test for categorical variables. Significantly different variables (*p* < 0.1) were further analyzed in the multinomial logistic regression of the latent classes.

Statistical analyses were conducted by IBM SPSS 23.0 (IBM Svenska AB, 16,492 Stockholm) and SAS 9.4 (SAS Institute, Cary, NC). A *P* < 0.05 was considered statistically significant.

### 2.5. Sensitivity analyses

To evaluate the robustness of our findings, sensitivity analyses were conducted by selecting participants with at least four follow-up visits in different years and participants who did not change their ART regimens during follow-up, respectively.

## 3. Results

### 3.1. Cohort description

In total, 1,572 patients tested positive for HIV were eligible for inclusion and were included in these analyses. Supplementary Figure 1 shows flow chart details. The cohort was predominately male (95.5%), infected by homosexual transmission (75.8%), and with a median age of 31 years (IQR 26–42) (Table 1). At the baseline, NNRTI regimens were used by 92.6%, PI regimens by 3.6%, and INSTI regimens by 3.9%. The two backbone drugs were mainly lamivudine (3TC) plus tenofovir (TDF) (83.8%) or 3TC plus AZT (12.8%). The median follow-up time for patients from the initiation of ART to the last available visit was 2.0 years (IQR 1.0–4.7). In total, 60% of participants had at least four measurements in different years. During follow-up, 1,926 (82.4%) patients maintained their initial treatment regimens, and 276 (17.6%) patients changed their regimens, with 110 (7.0%) patients having their core drug substituted from NNRTI to INSTI, 91 (5.8%) patients having their core drug substituted from NNRTI to PI, and 75 (4.8%) having other ART shifts.

**Table 1:** Characteristics of the study population (*n* = 1,572).

**Characteristics**	***n***	**%**	**Characteristics**	***n***	**%**
**Number of assessments**			≥1.7	522	33.2
2	271	17.2	**TC (mmol/l)**		
3	258	16.4	<5.2	1,442	91.7
4	214	13.6	≥5.2	130	8.3
5	238	15.1	**Route of infection**		
6–8	591	37.6	Homosexual transmission	1,192	75.8
**Gender**			Heterosexual transmission and others	380	24.2
Male	1,501	95.5	**CD4 cell count (cells/μl)**		
Female	71	4.5	<200	536	34.1
**Age (years)**			200–349	596	37.9
18–29	647	41.2	350–499	302	19.2
30–44	595	37.8	≥500	138	8.8
45–59	224	14.2	**HIV viral load (copies/ml)**		
≥60	106	6.7	Missing	1,090	69.3
**BMI (kg/m** ^**2**^ **)**			≤ 100,000	378	24.0
<18.5	102	6.5	>100,000	104	6.6
18.5–23.9	1,105	70.3	**Backbone drugs**		
≥24	365	23.2	3TC+TDF	1,317	83.8
**FPG (mmol/l)**			3TC+AZT	192	12.2
<6.1	1,241	78.9	Other	63	4.0
6.1–6.9	186	11.8	**Core drugs**		
≥7.0	145	9.2	NNRTIs	1,455	92.6
**TG (mmol/l)**			PIs	56	3.6
<1.7	1,050	66.8	INSTIs	61	3.9

BMI, body mass index; FPG, fasting plasma glucose; TG, triglyceride; TC, total cholesterol; HAART, highly active antiretroviral therapy; 3TC, lamivudin; TDF, tenofovir; AZT, zidovudine; NNRTIs, non-nucleoside reverse transcriptase inhibitors; PIs, protease inhibitors; INSTIs, integrase strand transfer inhibitors.

### 3.2. GMM model fitting results

First, by comparing model fit indices, the quadratic GCM was selected as the base model, which showed the best-fit indices (Supplementary Table 1). Then, the 2-classes GBTM to 9-classes GBTM were analyzed (Supplementary Table 2). Based on the VLMR and aLMR results, the 8-classes GBTM model was selected. Since the information criteria (IC) of GBTM models were gradually improved as the number of groups increased, the 8-classes LCGA models with different residual variance structures were allowed to be fitted, i.e., same over class but different across time (LCGA1), same over time but different over class (LCGA2), and different across time and over class (LCGA3) (29). Still, the 3-classes LCGA2 model was selected based on the VLMR and aLMR results and BIC values (Supplementary Table 3).

According to the IC behaviors of LCGA models, we then expanded the LCGA2 into GMMs by adding class-variant random effect variances stepwise, respectively. Finally, the GMM2 with class-variant random intercept and linear slope variance had lower BICs than others (Supplementary Table 3). The VLMR and aLMR results showed that the 3-classes model could be retained. After further eliminating the non-significant higher-order polynomial terms, the GMM4 model was settled on as the final selected model, with the lowest BIC value of −13,623.117 and with APPAs of 0.89, 0.76, and 0.80 for each class, respectively. Furthermore, the GMM4 model had a reasonable distribution of class memberships across the categories (7.2, 30.5, and 62.3%).

**Figure 1:**
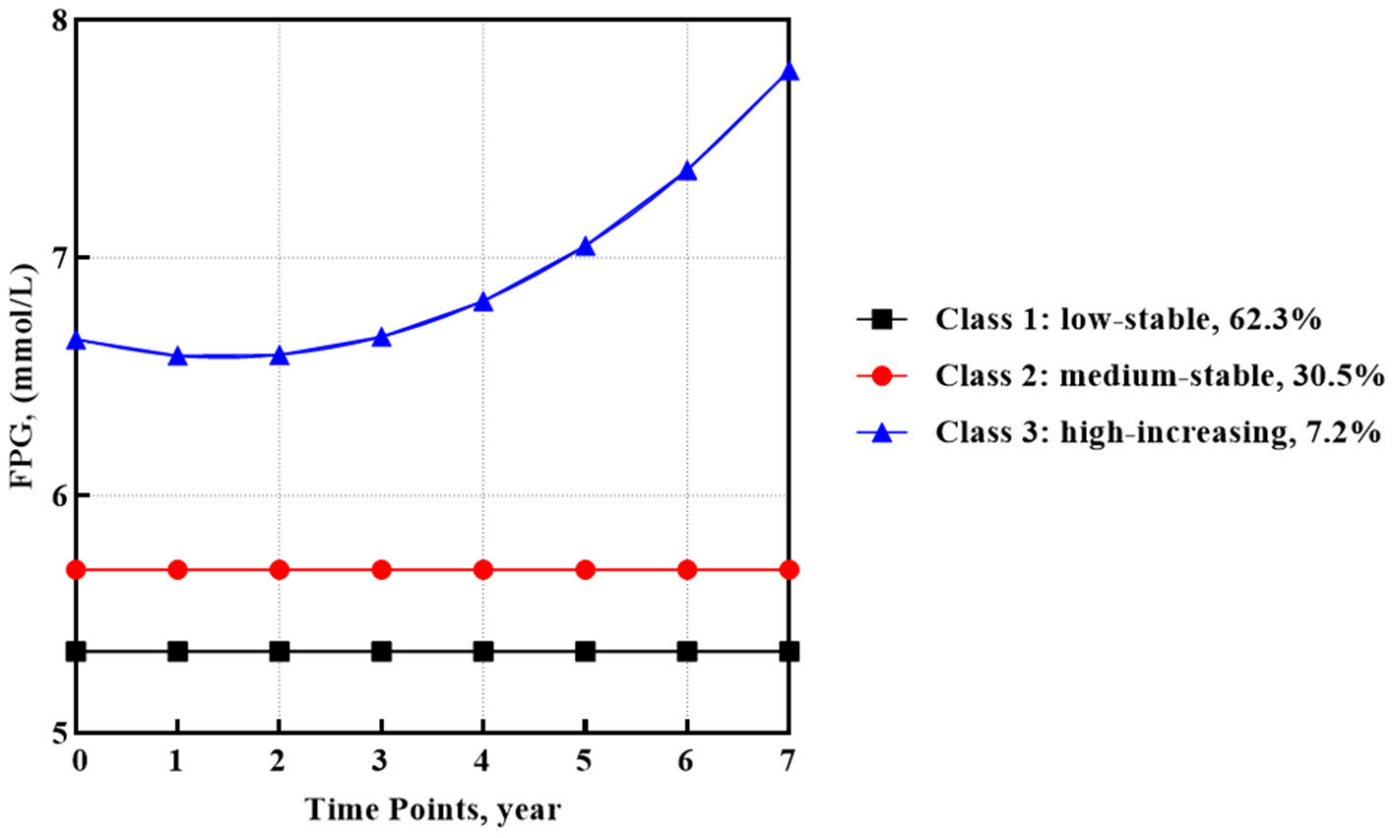
FPG trajectories after HAART initiation identified by GMM (*n* = 1,572).

In the GMM model for FPG, the best fit of the 3-classes model involved one quadratic and two intercept trajectories (Figure 1). Most participants (62.3%, *n* = 979) categorized into class 1 observed a low-stable FPG trajectory, which began around 5.34 mmol/l. In addition, class 2 containing 30.5% of participants (*n* = 479) showed a medium-stable FPG trajectory that began around 5.69 mmol/l. The rest of the participants (7.2%, *n* = 114) in class 3 observed a high-increasing FPG trajectory with the highest baseline mean FPG value of 6.66 mmol/l and a quadratic increase with a slope of 0.005 (SE = 0.002, *p* < 0.05).

The fitting of dual-trajectories for TG and TC followed the same modeling strategy as for FPG. Based on the selected quadratic GCM model, the 3-classes GMM1 model with a relaxed time-varying and class-varying residual and random intercept variance structures was ultimately settled on as the optimal model, with the lowest BIC value of −8,426.30 and APPAs of 0.85, 0.78, and 0.79 for each class, respectively (Supplementary Tables 4–6). Furthermore, the class membership for the GMM model was 13.7, 27.6, and 58.7% for each category.

**Figure 2:**
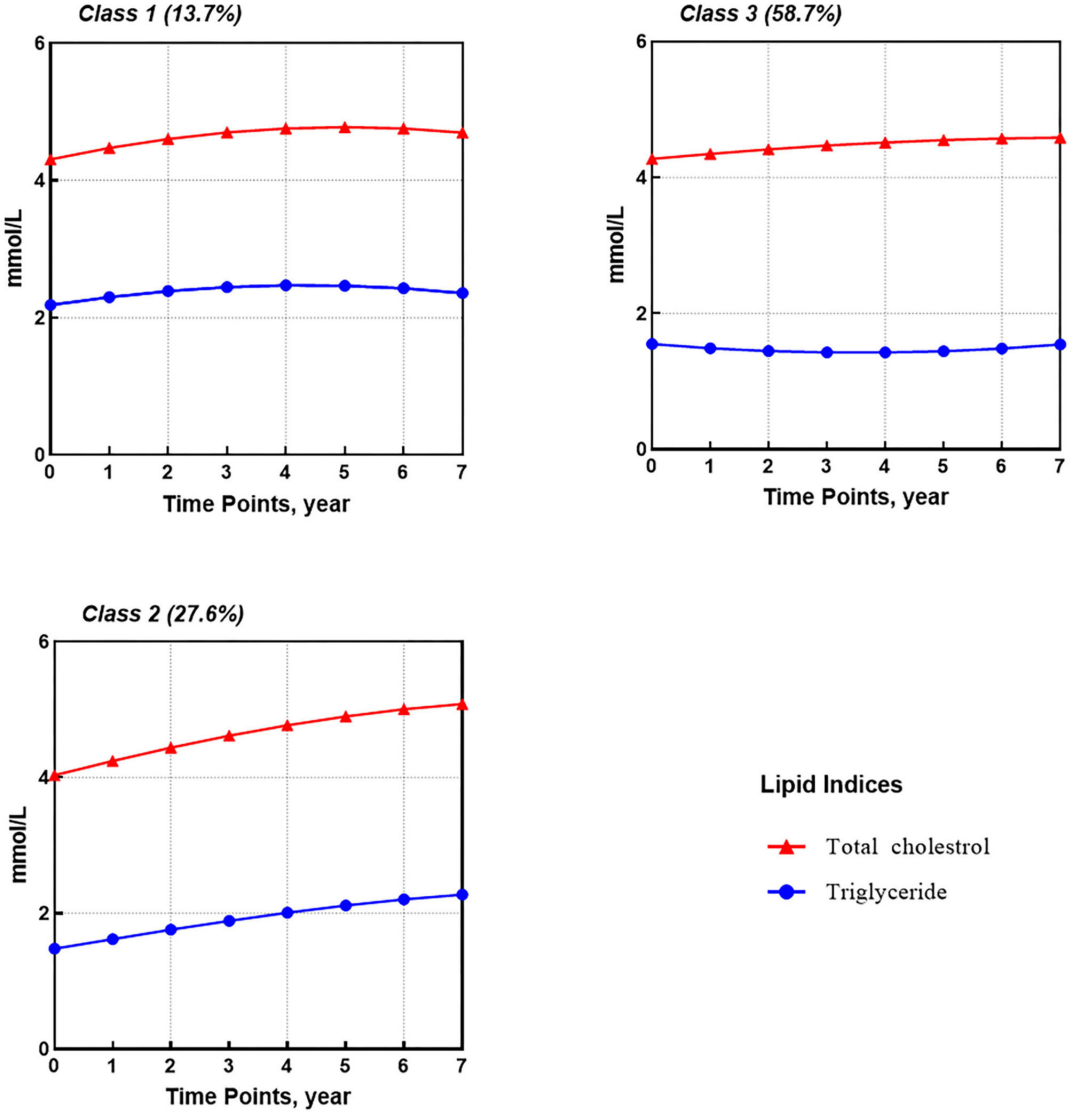
Dual-trajectories of TG and TC after HAART initiation identified by GMM (*n* = 1,572).

For TG and TC (Figure 2), three shared latent groups were identified: (1) high-slight increasing TG and TC (class 1, 13.7%); (2) low-rapid increasing TG and TC (class 2, 27.6%); and (3) medium-stable TC and slight-decreasing TG (class 3, 58.7%). Supplementary Table 7 provides parameter estimates for latent growth factors based on the best solution, i.e., the 3-classes GMM1.

### 3.3. Predictors of FPG trajectory subgroups

Table 2 shows the baseline characteristics of participants in different FPG trajectory subgroups. Compared to class 1, participants in classes 2 and 3 were featured by older age, higher TG and FPG at baseline, and use of non-TDF backbone drugs, while only participants in class 3 were characterized by baseline CD4 counts of <200 cells/μl and BMI of ≥18.5 kg/m^2^. No significant differences were found between classes 2 and 3 except for age and FPG levels at the baseline. In the multivariable model, age older than 30 years and high FPG were independently associated with classes 2 and 3 when compared with class 1. The initial use of AZT was independently associated with class 2 but marginally associated with class 3.

**Table 2:** Baseline characteristics of participants among three FPG trajectory subgroups.

**Characteristics**	**Class 1** **(*n* = 979)**	**Class 2** **(*n* = 479)**	**Class 3** **(*n* = 114)**	***P* ^a^**	**adjusted OR**^**b**^ **(95%CI)**	

					**Class 2** **(*****n*** = **479)**	**Class 3** **(*****n*** = **114)**
**Sex**				0.372		
Male	937 (95.7)	453 (94.6)	111 (97.4)			
Female	42 (4.3)	26 (5.4)	3 (2.6)			
**Age (years)**				<0.001		
18–29	502 (51.3)	129 (26.9)	16 (14.0)		1.00	1.00
30–44	361 (36.9)	196 (40.9)	38 (33.3)		2.07 (1.57–2.71)	2.58 (1.37–4.87)
45–59	80 (8.2)	102 (21.3)	42 (36.8)		4.64 (3.16–6.80)	8.94 (4.44–17.98)
≥60	36 (3.7)	52 (10.9)	18 (15.8)		4.72 (2.83–7.87)	7.79 (3.29–18.44)
**BMI (kg/m** ^2^ **)**				<0.001		
≥24	200 (20.4)	113 (23.6)	52 (45.6)		1.00	1.00
18.5–23.9	712 (72.7)	338 (70.6)	55 (48.2)		1.06 (0.79–1.41)	0.47 (0.30–0.75)
<18.5	67 (6.8)	28 (5.8)	7 (6.1)		1.19 (0.70–2.03)	0.94 (0.36–2.47)
**FPG (mmol/l)**				<0.001		
<6.1	705 (72.0)	269 (56.2)	27 (23.7)		1.00	1.00
6.1–6.9	231 (23.6)	155 (32.4)	40 (35.1)		1.60 (1.13–2.27)	3.15 (1.75–5.64)
≥7.0	43 (4.4)	55 (11.5)	47 (41.2)		2.53 (1.62–3.93)	14.00 (8.04–24.37)
**TG (mmol/l)**				<0.001		
<1.7	689 (70.4)	304 (63.5)	57 (50.0)		1.00	1.00
≥1.7	290 (29.6)	175 (36.5)	57 (50.0)		1.13 (0.88–1.46)	1.49 (0.96–2.34)
**TC (mmol/l)**				0.843		
<5.2	900 (91.9)	439 (91.6)	103 (90.4)			
≥5.2	79 (8.1)	40 (8.4)	11 (9.6)			
**Route of infection**				<0.001		
Homosexual transmission	779 (79.6)	340 (71.0)	73 (64.0)		1.00	1.00
Heterosexual transmission and others	200 (20.4)	139 (29.0)	41 (36.0)		1.00 (0.75–1.33)	1.08 (0.66–1.77)
**CD4 cell count (cells/**μ**l)**				0.043		
<200	309 (31.6)	176 (36.7)	51 (44.7)		1.00	1.00
200–349	377 (38.5)	186 (38.8)	33 (28.9)		1.09 (0.83–1.43)	0.68 (0.40–1.15)
350–499	200 (20.4)	82 (17.1)	20 (17.5)		1.01 (0.72–1.42)	1.02 (0.55–1.91)
≥500	93 (9.5)	35 (7.3)	10 (8.8)		0.84 (0.53–1.33)	0.78 (0.34–1.79)
**HIV viral load (copies/ml)**				0.083		
Missing	657 (67.1)	350 (73.1)	83 (72.8)		1.00	1.00
≤ 100,000	259 (26.5)	96 (20.0)	23 (20.2)		0.77 (0.58–1.03)	0.75 (0.43–1.31)
>100,000	63 (6.4)	33(6.9)	8 (7.0)		0.99 (0.62–1.57)	0.93 (0.38–2.27)
**Backbone drugs**				<0.001		
3TC+TDF	96 (9.8)	74 (15.4)	22 (19.3)		1.00	1.00
3TC+AZT	845 (86.3)	388 (81.0)	84 (73.7)		1.44 (1.02–2.05)	1.73 (0.95–3.15)
Others	38 (3.9)	17 (3.5)	8 (7.0)		0.71 (0.38–1.33)	1.23 (0.49–3.05)
**Core drugs**				0.725		
NNRTIs	902 (92.1)	446 (93.1)	107 (93.9)			
PIs	36 (3.7)	18 (3.8)	2 (1.8)			
INSTIs	41 (4.2)	15 (3.1)	5 (4.4)			

BMI, body mass index; FPG, fasting plasma glucose; TG, triglyceride; TC, total cholesterol; HAART, highly active antiretroviral therapy; 3TC, lamivudin; TDF, tenofovir; AZT, zidovudine; NNRTIs, non-nucleoside reverse transcriptase inhibitors; PIs, protease inhibitors; INSTIs, integrase strand transfer inhibitors; CI, confidence interval; OR, odds ratio; Class 1, low-stable FPG group; Class 2, medium-stable FPG group; Class 3, high-increasing FPG group.

^a^P-values were calculated by chi-square test or Fisher's exact test.

^b^ORs derived from log-odds estimates for the risk factors for each group relative to Class 1.

### 3.4. Predictors of TG and TC dual-trajectory subgroups

Table 3 shows the baseline characteristics of participants in different TG and TC dual-trajectory subgroups. In comparison with class 3, which showed medium-stable TC and slight-decreasing TG trajectories, participants in class 1 with high-slight increasing TG and TC were older, overweight, or obese, with baseline CD4 counts of <200 cells/μl, high TG, and initial use of AZT and PIs.

**Table 3:** Baseline characteristics of participants among three TG and TC dual-trajectory subgroups.

**Characteristics**	**Class 1** **(*n* = 216)**	**Class 2** **(*n* = 433)**	**Class 3** **(*n* = 923)**	***P^a^***	**adjusted OR**^**b**^ **(95%CI)**	

					**Class 1** **(*****n*** = **216)**	**Class 2** **(*****n*** = **433)**
**Sex**				0.098		
Male	203 (94.0)	421 (97.2)	877 (95.0)		1.00	1.00
Female	13 (6.0)	12 (2.8)	46 (5.0)		0.88 (0.44–1.77)	0.58 (0.29–1.16)
**Age (years)**				<0.001		
18–29	61 (28.2)	181 (41.8)	405 (43.9)		1.00	1.00
30–44	89 (41.2)	173 (40.0)	333 (36.1)		1.45 (1.00–2.11)	1.13 (0.86–1.47)
45–59	47 (21.8)	51 (11.8)	126 (13.7)		1.64 (1.02–2.64)	0.87 (0.58–1.29)
≥60	19 (8.8)	28 (6.5)	59 (6.4)		1.31 (0.69–2.50)	0.98 (0.58–1.66)
**BMI (kg/m** ^2^ **)**				0.021		
≥24	11 (5.1)	20 (4.6)	71 (7.7)		1.00	1.00
18.5–23.9	140 (64.8)	313 (72.3)	652 (70.6)		0.80 (0.56–1.14)	0.97 (0.73–1.29)
<18.5	65 (30.1)	100 (23.1)	200 (21.7)		0.63 (0.30–1.30)	0.58 (0.33–1.02)
**TG (mmol/l)**				<0.001		
<1.7	111 (51.4)	283 (65.4)	656 (71.1)		1.00	1.00
≥1.7	105 (48.6)	150 (34.6)	267 (28.9)		2.04 (1.49–2.80)	1.26 (0.98–1.62)
**TC (mmol/l)**				0.317		
<5.2	199 (92.1)	404 (93.3)	839 (90.9)			
≥5.2	17 (7.9)	29 (6.7)	84 (9.1)			
**Route of infection**				0.003		
Homosexual transmission	145 (67.1)	342 (79.0)	705 (76.4)		1.00	1.00
Heterosexual transmission and others	71 (32.9)	91 (21.0)	218 (23.6)		1.37 (0.95–1.99)	0.93 (0.68–1.27)
**CD4 cell count (cells/**μ**l)**				0.056		
<200	93 (43.1)	150 (34.6)	293 (31.7)		1.00	1.00
200–349	68 (31.5)	155 (35.8)	373 (40.4)		0.66 (0.46–0.95)	0.83 (0.63–1.10)
350–499	36 (16.7)	90 (20.8)	176 (19.1)		0.78 (0.49–1.22)	1.06 (0.76–1.48)
≥500	19 (8.8)	38 (8.8)	81 (8.8)		0.85 (0.47–1.51)	0.99 (0.63–1.54)
**HIV viral load (copies/ml)**				0.002		
Missing	154 (71.3)	322 (74.4)	614 (66.5)		1.00	1.00
≤ 100,000	43 (19.9)	80 (18.5)	255 (27.6)		0.73 (0.49–1.08)	0.63 (0.47–0.84)
>100,000	19 (8.8)	31 (7.2)	54(5.9)		1.36 (0.77–2.41)	1.11 (0.70–1.78)
**Backbone drugs**				<0.001		
3TC+TDF	164 (75.9)	361 (83.4)	792 (85.8)		1.00	1.00
3TC+AZT	37 (17.1)	62 (14.3)	93 (10.1)		1.72 (1.11–2.66)	1.41 (0.99–2.02)
Others	15 (6.9)	10 (2.3)	38 (4.1)		1.91 (0.87–4.19)	0.82 (0.35–1.90)
**Core drugs**				0.019		
NNRTIs	190 (88.0)	407 (94.0)	858 (93.0)		1.00	1.00
PIs	14 (6.5)	16 (3.7)	26 (2.8)		2.36 (1.17–4.74)	1.28 (0.67–2.44)
INSTIs	12 (5.6)	10 (2.3)	39 (4.2)		0.94 (0.41–2.16)	0.67 (0.29–1.54)

BMI, body mass index; FPG, fasting plasma glucose; TG, triglyceride; TC, total cholesterol; HAART, highly active antiretroviral therapy; 3TC, lamivudin; TDF, tenofovir; AZT, zidovudine; NNRTIs, non-nucleoside reverse transcriptase inhibitors; PIs, protease inhibitors; INSTIs, integrase strand transfer inhibitors; CI, confidence interval; OR, odds ratio; Class 1, low-stable FPG group; Class 2, medium-stable FPG group; Class 3, high-increasing FPG group.

^a^P-values were calculated by chi-square test or Fisher's exact test.

^b^ORs derived from log-odds estimates for the risk factors for each group relative to Class 1.

In the multivariable model (Table 3), age between 30 and 60 years, with high TG, the initial use of AZT and PIs were independently positively associated with class 1 when compared with class 3, whereas CD4 counts between 200 and 350 cells/μl had a negative association with class 1. HIV load of ≤ 100,000 copies/ml was independently negatively associated with class 2, whereas high TG and initial use of AZT had a marginally significant association with class 2.

### 3.5. Sensitivity analyses

No substantially different results were observed when limiting to participants with at least four measurements in different years or patients who maintained their initial ART regimens during follow-up in the outcomes of the GMM models, and the interpretation of the trajectory curves remained similar (Supplementary Figures 2–5).

## 4. Discussion

Our study illustrates how GMM can be used to identify subgroups of longitudinal trajectories of plasma glucose and lipid levels in patients infected with HIV receiving HAART without any preconceived assumptions about the number and type of longitudinal profiles. Three FPG trajectory subgroups and three TG and TC dual-trajectory subgroups were identified, demonstrating heterogeneity in longitudinal changes in plasma glucose and lipid levels.

Although the overall FPG measurements showed an increasing tendency, most patients tested positive for HIV maintained stable FPG during follow-up. Few of the patients observed a strong non-linear increase in FPG, with a rising growth rate in the last few years of follow-up after a slight decrease in the first year. A recent study also showed that FPG declined and insulin increased during 12 months of ART, indicating that there might be an improvement in insulin secretion and health (30). In addition, some studies have shown that the longer the duration of HAART, the higher the risk of diabetes (31, 32), reflecting the possible cumulative effects of HIV infection or ART on diabetes.

It has been suggested that older age and high FPG levels were risk factors for diabetes and pre-diabetes (10). Similarly, in this study, age ≥30 years and high FPG were independent risk factors for medium-stable and high-increasing FPG trajectories. In addition, the D:A:D study demonstrated that diabetes risk among patients tested positive for HIV on ART increased linearly with increasing BMI (33). However, our research manifested that patients with overweight or obese had a higher risk of a high-increasing FPG trajectory than those with normal weight but had no statistically significant difference when compared with those underweight, presumably related to reverse causalities, such as a negative association between current smoking and BMI (34), which is an important contributor to the risk of diabetes (35). The association between baseline CD4 levels and different trajectories of FPG disappeared in the multivariable model. Some studies also did not reveal an association between CD4 levels and diabetes (36). In contrast, other studies demonstrated a negative association (37, 38), speculating that the improvement of immune system functionality might enhance glycemic control by easing the inflammatory response and oxidative stress to viral infection.

Initial use of AZT remained an independent risk factor for medium-stable FPG compared to TDF after adjusting for BMI and TG levels, consistent with the results of several studies (39, 40). The D:A:D research revealed that AZT remained an independent risk factor for diabetes after adjustment for lipids and lipodystrophy, suggesting that AZT may directly contribute to insulin resistance through mitochondrial toxicity (39).

In our research, both TG and TC levels increased during follow-up. Some previous evidence revealed that, before the initiation of ART, HIV infection resulted in lower TC, while increases in TC were observed after initiating ART (41), with feasible mechanisms being a reduction of systemic inflammation with virologic suppression (42). Unlike TC, TG levels were often elevated, related to reduced TG clearance, and the enhanced hepatic release of very-low-density lipoprotein (43). Interestingly, our research identified a stable TC and U-shaped slight-decreasing TG trajectory subgroup and a subgroup with the lowest TG and TC levels at the baseline but significantly increased during follow-up, indicating that lipid changing patterns were heterogeneous and might depend on multifactor such as the use of specific antiretroviral medications and lipid-lowering medications, genetic traits, and lifestyle factors.

Compared with individuals in class 3 presenting with optimal levels of these lipids, those in class 1 with initially high-increasing and slight-increasing TG and TC trajectories were characterized as middle-aged, hypertriglyceridemia at the baseline, initial use of AZT and PIs, and poorer health status at HAART initiation (i.e., CD4 counts of <200 cells/μl). A systematic review suggested that CD4 counts could be correlated with a greater chance of lipid changes (42). But no consistent conclusion has been reached on the direction and strength of the correlation between the CD4 counts and the lipid profiles (44, 45).

The class of antiretroviral drugs most frequently associated with dyslipidemia were probably the older generation PIs (11, 46), which can cause significant increases in TG, TC, and LDL-C levels. Similarly, our research found that the use of PI dominated by the older generation (i.e., LPV/r) was associated with the high-slight increasing lipid trajectory. Mechanisms of PIs producing modifications of lipid profile remain to be fully articulated. Putative mechanisms were that the sterol regulatory element–binding protein (SREBP)-1c regulating genes required for fatty acid metabolism, *de novo* cholesterol synthesis, and clearance of TG-rich and cholesterol-rich lipoproteins were inhibited by older generation PIs (46).

In addition, our results were comparable with previous research reporting that the AZT-containing regimen remained a risk factor for deteriorating TG and TC trajectories compared to the TDF-containing regimen (42, 46). TDF has been evidenced to have a lipid-lowering effect (47, 48) and was recommended as a backbone drug of first-line treatment (49). In contrast, AZT has been proven to be associated with lipid abnormalities (50). High ROS concentrations, associated with severe mitochondrial dysfunction produced by NRTIs, especially d4T and AZT, inhibited the expression of the adipogenic factor mitochondrial DNA (mtDNA) polymerase-⋎ and induced cell apoptosis, which could result in lipoatrophy and increase in free fatty acids (51).

The present study has the following strengths. First, the GMM approach, we used, had unique advantages over traditional analysis in describing the developmental process and classifying participants into diverse, mutually exclusive groups. Second, the dual-trajectory analysis used improved the accuracy of individual-specific probabilities of given group membership by integrating the interrelationship between both lipids. Despite these advantages, this study has limitations. First, some data, including diet, physical activity, and lipid-lowering medicine, were not collected, which could have been critical to the topic. Therefore, caution should be exercised in interpreting the present results. Second, this study was based on data from large public sector government HIV clinics in China, where most participants were patients with NNRTI-based regimens, male, and Asiatic. Thus, sample representativeness might be limited. Finally, given that we hope but did not have an opportunity to estimate the effect of FPG, TG, and TC trajectories on the hazard of CVD, more studies with large populations are needed to explore the association between the longitudinal trajectories of metabolic indicators and CVD incidence risk.

## 5. Conclusion

This study provides new information on the longitudinal development of FPG in patients tested positive for HIV on HAART with distinct trajectories of low-stable, medium-stable, and high-increasing classes. At the same time, three TG and TC joint development patterns were found, namely, high-slight increasing, low-rapid increasing TG and TC trajectories, and medium-stable TC and slight-decreasing TG trajectories. In brief, older age, poorer metabolic and immune status at the baseline, and initial use of some specific antiretrovirals (i.e., AZT and PIs) helped to identify the class with increasing glucose and lipid metabolism trajectories. Further research into the biological mechanisms explaining these underlying patterns of change is needed to discover and guide the future management of abnormal glucose and lipid metabolism in patients tested positive for HIV.

## Data availability statement

The original contributions presented in the study are included in the article/Supplementary material, further inquiries can be directed to the corresponding author.

## Ethics statement

The studies involving human participants were reviewed and approved by the Ethics Committee of Pudong New District Center for Disease Control and Prevention. Written informed consent for participation was not required for this study in accordance with the national legislation and the institutional requirements.

## Author contributions

JL: methodology, data analysis, and writing–original draft. XX: data analysis and writing–review. PC: methodology and writing–review. ZN: conceptualization and writing–review. SX: resources, writing–review, and supervision. All authors contributed to the article and approved the submitted version.
