# Corrigendum: Distinct patterns of fasting plasma glucose and lipid profile levels over time in adults tested positive for HIV on HAART in Shanghai, China, revealed using growth mixture models

**DOI:** 10.3389/fmed.2023.1169491

**Published:** 2023-03-07

**Authors:** Jingjing Lang, Xin Xin, Panpan Chen, Zhen Ning, Shaotan Xiao

**Affiliations:** ^1^School of Public Health, Fudan University, Shanghai, China; ^2^Pudong New Area Center for Disease Control and Prevention, Shanghai, China; ^3^Pudong Institute of Preventive Medicine, Fudan University, Shanghai, China; ^4^Shanghai Center for Disease Control and Prevention, Shanghai, China

**Keywords:** HIV, HAART, growth mixture model, fasting plasma glucose, lipid

In the published article, there was an error in Figure 1 as published. Figure 2 was incorrectly uploaded as Figure 1. Also, there was a mistake in the caption for Figure 1 as published. The corrected Figure 1 and its caption appear below.

**Figure 1:**
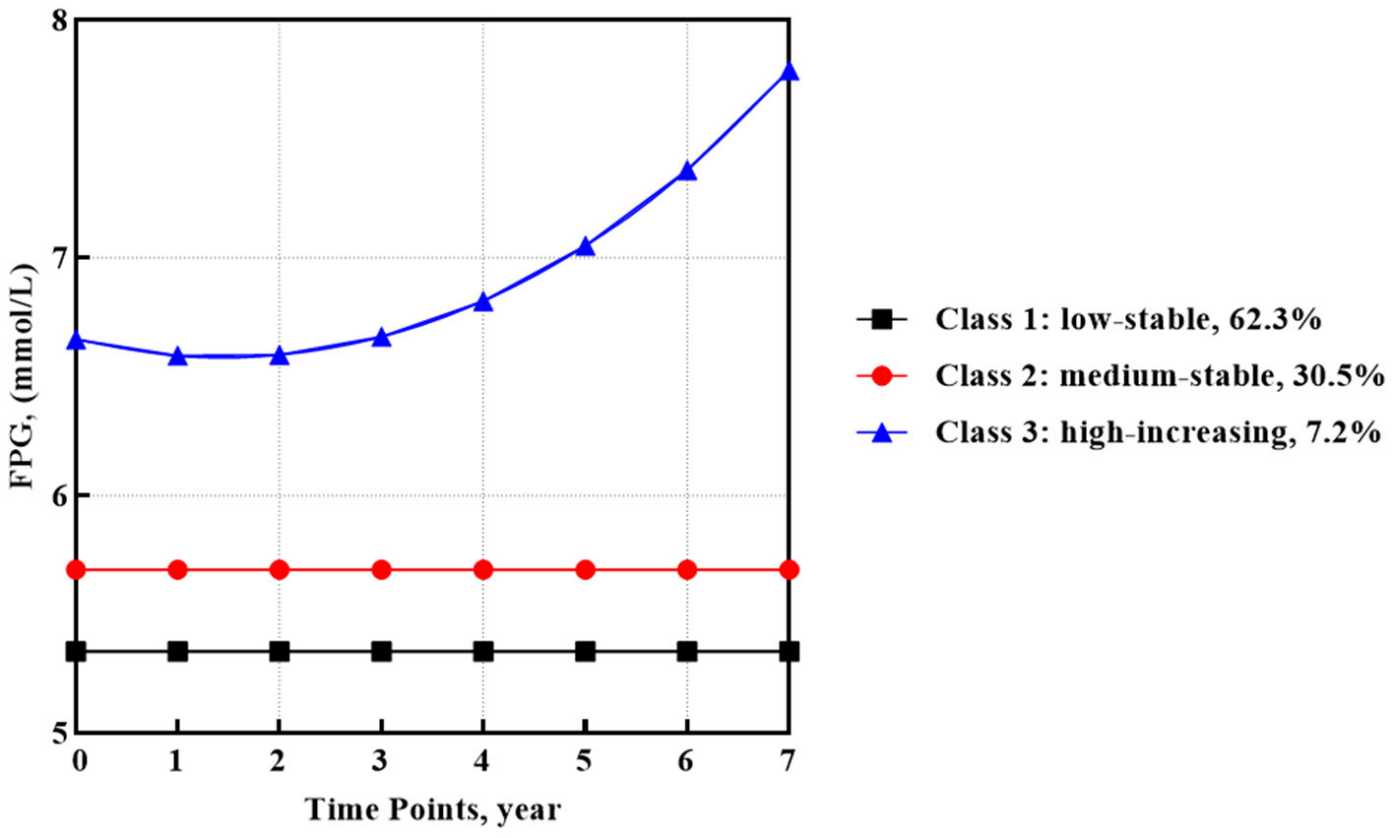
FPG trajectories after HAART initiation identified by GMM (*n* = 1,572).

In the published article, there was an error. **The proportions of members contained in FPG trajectory subgroups were incorrectly written**.

A correction has been made to **Results**, ***GMM model fitting results***. This sentence previously stated:

“Most participants (51.8%, *n* = 979) categorized into class 1 observed a low-stable FPG trajectory, which began around 5.34 mmol/l. In addition, class 2 containing 38.9% of participants (*n* = 479) showed a medium-stable FPG trajectory that began around 5.69 mmol/l. The rest of the participants (9.6%, *n* = 114) in class 3 observed a high-increasing FPG trajectory with the highest baseline mean FPG value of 6.66 mmol/l and a quadratic increase with a slope of 0.005 (SE = 0.002, *p* < 0.05).”

The corrected sentence appears below:

“Most participants (62.3%, *n* = 979) categorized into class 1 observed a low-stable FPG trajectory, which began around 5.34 mmol/l. In addition, class 2 containing 30.5% of participants (*n* = 479) showed a medium-stable FPG trajectory that began around 5.69 mmol/l. The rest of the participants (7.2%, *n* = 114) in class 3 observed a high-increasing FPG trajectory with the highest baseline mean FPG value of 6.66 mmol/l and a quadratic increase with a slope of 0.005 (SE = 0.002, *p* < 0.05).”

The authors apologize for these errors and state that this does not change the scientific conclusions of the article in any way. The original article has been updated.

