# Supplementary material for: Distinct patterns of fasting plasma glucose and lipid profile levels over time in adults tested positive for HIV on HAART in Shanghai, China, revealed using growth mixture models
Source: Front Med (Lausanne). 2023 Jan 17;9:1071431. doi: 10.3389/fmed.2022.1071431 (PMC9887111; doi:10.3389/fmed.2022.1071431)

Supplemental Figure 1. Study inclusion criteria.

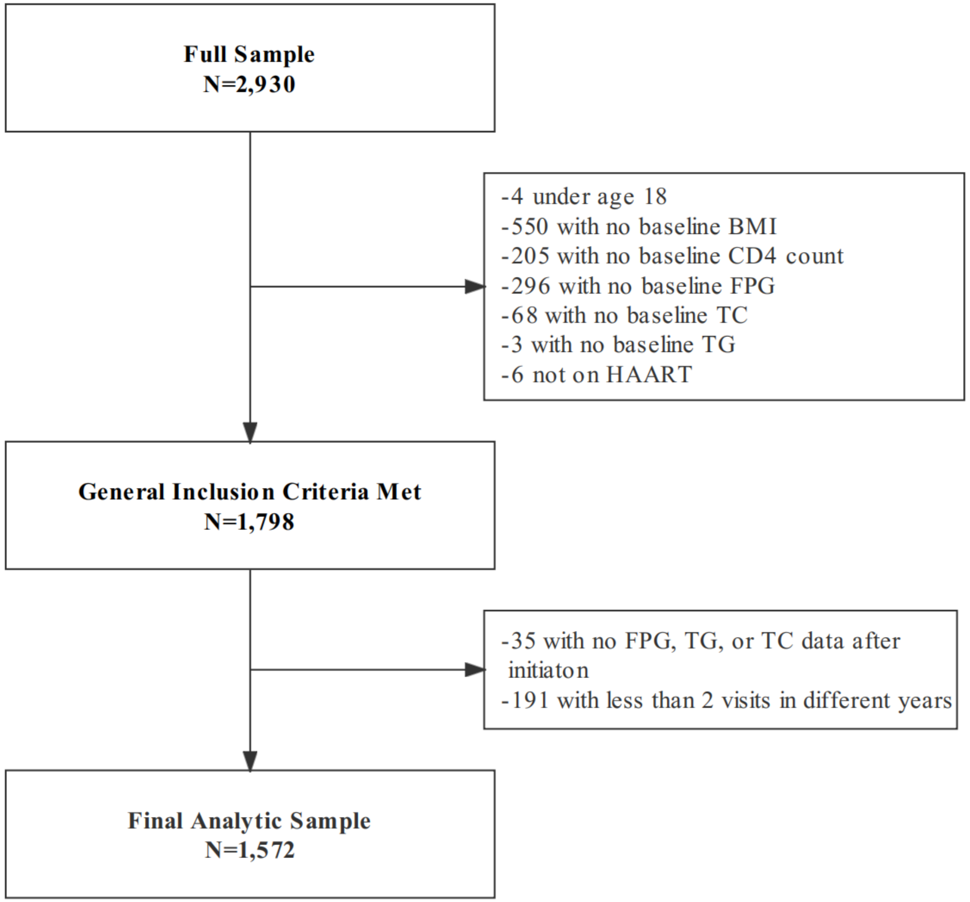

Supplementary Table 1. The model fit indices of three LGCM models for FPG.

Model	χ^2	RMSEA (90% CI)	CFI	TLI	SRMR
LGCM01 Linear	68.787***	0.028 (0.019-0.037)	0.990	0.991	0.080
LGCM02 Quadratic★	46.797***	0.022 (0.010-0.032)	0.995	0.995	0.073
LGCM03 Latent basis	44.409***	0.022 (0.011-0.033)	0.995	0.994	0.067

RMSEA = Root mean square error of approximation; CI = Confidence interval; CFI = Comparative fit index; TLI = Tucker-Lewis index; SRMR = Standardized root mean square residual. *** p <0.001, ** p <0.01, * p <0.05. ★ indicates the optimal solution based on the model fit indices.

Supplementary Table 2. The model fit indices of GBTM models for FPG.

Model	GBTM2	GBTM3	GBTM4	GBTM5	GBTM6	GBTM7	GBTM8★	GBTM9
**Group**	2	3	4	5	6	7	8	9
**LL**	5378.107	5805.999	6042.86	6141.143	6200.036	6259.089	6301.605	6344.749
**AIC**	-10740.214	-11587.998	-12053.720	-12242.286	-12352.071	-12462.178	-12539.211	-12617.498
**BIC**	-10697.333	-11523.677	-11967.959	-12135.084	-12223.429	-12312.096	-12367.687	-12424.534
**sABIC**	-10722.747	-11561.798	-12018.787	-12198.62	-12299.672	-12401.045	-12469.344	-12538.898
**Lowest APPA**	0.953	0.857	0.841	0.798	0.787	0.783	0.799	0.729
**Scaled Entropy**	0.961	0.782	0.787	0.744	0.743	0.759	0.776	0.767
**VLMR p-value**	0.0213	<0.0001	<0.0001	<0.0001	<0.0001	<0.0001	<0.0001	0.1389
**aLMR p-value**	0.0235	<0.0001	<0.0001	<0.0001	<0.0001	<0.0001	<0.0001	0.1394

LL = Log-likelihood; AIC = Akaike information criterion; BIC = Bayesian information criterion; sABIC = Sample size adjusted BIC; APPA = average posterior probability of assignment; VLMR = Vuong-Lo-Mendell-Rubin likelihood ratio test; aLMR = adjusted Lo-Mendell–Rubin likelihood ratio test. ★ indicates the optimal solution based on the model fit indices.

Supplementary Table 3. The model fit indices of LCGA and GMM models for FPG.

Model	LCGA1	LCGA2	LCGA3	LCGA2 (2 classes)	LCGA2 (3 classes)	LCGA2 (4 classes)	GMM1	GMM2	GMM3	GMM4★
**Group**	8	8	8	2	3	4	3	3	3	3
**Specification**	Same residual variance over class, different over time	Same residual variance over time, different over class	Different residual variance over time, different over class	Same residual variance over time, different over class	Same residual variance over time, different over class	Same residual variance over time, different over class	Same residual variance over time, different over class, class-variant random intercept variance	Same residual variance over time, different over class, class-variant random intercept, and liner slope variance	Same residual variance over time, different over class, class-variant random intercept, liner slope, and quadratic slope variance	Same residual variance over time, different over class, class-variant random intercept, and liner slope variance, all fixed effects significant
**LL**	6333.073	6803.260	6874.985	5831.281	6346.424	6522.282	6828.586	6876.010	6888.778	6874.120
**AIC**	-12588.147	-13528.520	-13559.971	-11644.562	-12664.848	-13006.564	-13623.172	-13710.021	-13725.556	-13714.239
**BIC**	-12379.103	-13319.476	-13050.761	-11596.321	-12589.807	-12904.722	-13532.051	-13597.459	-13586.193	-13623.117
**sABIC**	-12502.997	-13443.371	-13352.555	-11624.912	-12634.282	-12965.081	-13586.056	-13664.171	-13668.790	-13677.123
**Lowest APPA**	0.740	0.645	0.674	0.926	0.868	0.764	0.754	0.758	0.762	0.756

**Scaled Entropy**	0.755	0.651	0.662	0.870	0.747	0.702	0.551	0.562	0.572	0.556
**VLMR p-value**	0.005	0.432	0.346	<0.0001	<0.0001	0.2804	0.030	<0.0001	0.238	<0.0001
**aLMR p-value**	0.005	0.440	0.348	<0.0001	<0.0001	0.2864	0.032	<0.0001	0.238	<0.0001

LL, Log-likelihood; AIC, Akaike information criterion; BIC, Bayesian information criterion; sABIC, Sample size adjusted BIC; APPA, average posterior probability of assignment; VLMR, Vuong-Lo-Mendell-Rubin likelihood ratio test; aLMR, adjusted Lo-Mendell–Rubin likelihood ratio test. ★ indicates the optimal solution based on the model fit indices.

Supplementary Table 4. The model fit indices of three LGCM models for TG and TC.

Model	χ^2	RMSEA (90% CI)	CFI	TLI	SRMR
LGCM01 Linear	940.896***	0.065 (0.061-0.069)	0.951	0.952	0.154
LGCM02 Quadratic★	502.601***	0.048 (0.044-0.052)	0.977	0.974	0.051
LGCM03 Latent basis	811.313***	0.064 (0.060-0.068)	0.958	0.955	0.116

RMSEA, Root mean square error of approximation; CI, Confidence interval; CFI, Comparative fit index; TLI, Tucker-Lewis index; SRMR, Standardized root mean square residual. *** p <0.001, ** p <0.01, * p <0.05. ★ indicates the optimal solution based on the model fit indices.

Supplementary Table 5. The model fit indices of GBTM models for TG and TC.

Model	GBTM2	GBTM3	GBTM4	GBTM5★	GBTM6
**Group**	2	3	4	5	6
**LL**	-3818.153	-2242.402	-1533.597	-1174.787	-1010.495
**AIC**	7664.306	4526.804	3123.194	2419.574	2104.990
**BIC**	7739.347	4639.366	3273.277	2607.178	2330.114
**sABIC**	7694.872	4572.653	3184.327	2495.991	2196.689
**Lowest APPA**	0.971	0.943	0.915	0.885	0.869
**Scaled Entropy**	0.919	0.895	0.878	0.858	0.844
**VLMR p-value**	<0.0001	0.0480	<0.0001	<0.0001	0.2398
**aLMR p-value**	<0.0001	0.0501	<0.0001	<0.0001	0.2398

LL, Log-likelihood; AIC, Akaike information criterion; BIC, Bayesian information criterion; sABIC, Sample size adjusted BIC; APPA, average posterior probability of assignment; VLMR, Vuong-Lo-Mendell-Rubin likelihood ratio test; aLMR, adjusted Lo-Mendell–Rubin likelihood ratio test. ★ indicates the optimal solution based on the model fit indices.

Supplementary Table 6. The model fit indices of LCGA and GMM models for TG and TC.

Model	LCGA1	LCGA2	LCGA3	LCGA3 (2 classes)	LCGA3 (3 classes)	LCGA3 (4 classes)	GMM1	GMM1 ★ (3 classes)
**Group**	5	5	5	2	3	4	4	3
**Specification**	Same residual variance over class, different over time	Same residual variance over time, different over class	Different residual variance over time, different over class	Different residual variance over time, different over class	Different residual variance over time, different over class	Different residual variance over time, different over class	Different residual variance over time and class, class-variant random intercept variance	Different residual variance over time and class, class-variant random intercept variance
**LL**	1070.982	-1010.054	1363.619	-1372.050	773.009	-70.583	4620.015	4489.154
**AIC**	-2041.964	2098.109	-2499.237	2834.100	-1364.019	277.166	-9040.029	-8828.307
**BIC**	-1773.959	2307.153	-1888.185	3075.305	-876.249	641.653	-8504.019	-8426.3
**sABIC**	-1932.798	2183.258	-2250.339	2932.350	-1165.337	425.632	-8821.697	-8664.558
**Lowest APPA**	0.892	0.898	0.892	0.953	0.911	0.918	0.749	0.775
**Scaled Entropy**	0.866	0.861	0.874	0.857	0.878	0.866	0.569	0.565
**VLMR p-value**	0.2398	0.4285	0.1861	<0.0001	0.0007	0.0002	0.0886	<0.0001
**aLMR p-value**	0.2398	0.4343	0.1873	<0.0001	0.0007	0.0002	0.0909	<0.0001

LL, Log-likelihood; AIC, Akaike information criterion; BIC, Bayesian information criterion; sABIC, Sample size adjusted BIC; APPA, average posterior probability of assignment; VLMR, Vuong-Lo-Mendell-Rubin likelihood ratio test; aLMR, adjusted Lo-Mendell–Rubin likelihood ratio test. ★ indicates the optimal solution based on the model fit indices.

Supplementary Table 7. The model fit indices of LCGA and GMM models for TG and TC (n =1572).

Group	TG			TC		
	Intercept	Slope	Quadratic	Intercept	Slope	Quadratic
**High-slow increasing TG and TC**	0.782***	0.057	-0.007	1.461***	0.041***	-0.004*
**Low-rapid increasing TG and TC**	0.438***	-0.047***	0.007***	1.452***	0.019***	-0.001***
**Medium-stable TC and slight-decreasing TG**	0.391***	0.097***	-0.005***	1.395***	0.054***	-0.003***

TG, triglyceride; TC, total cholesterol; *** p <0.001, ** p <0.01, * p <0.05.

Supplemental Figure 2. FPG trajectories identified by GMM among participants with at least four measurements in different years (n =1044).

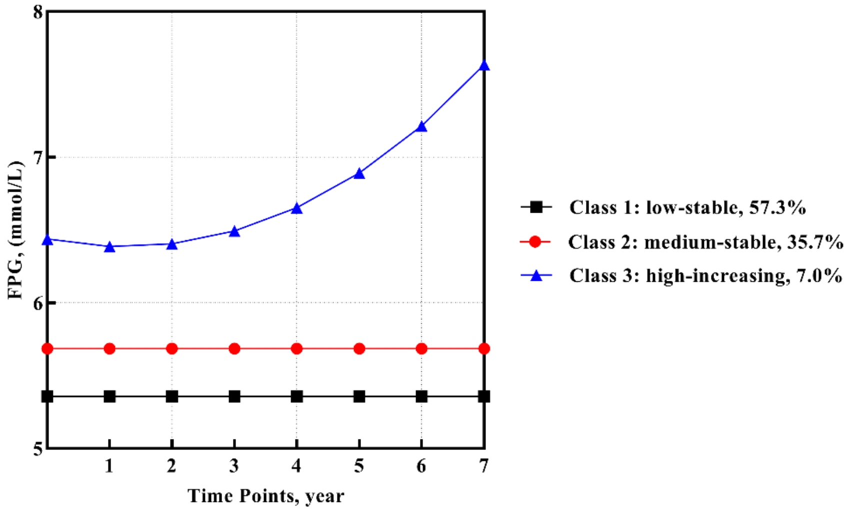

Supplemental Figure 3. Dual-trajectories of TG and TC identified by GMM among participants with at least four measurements in different years (n =1044).

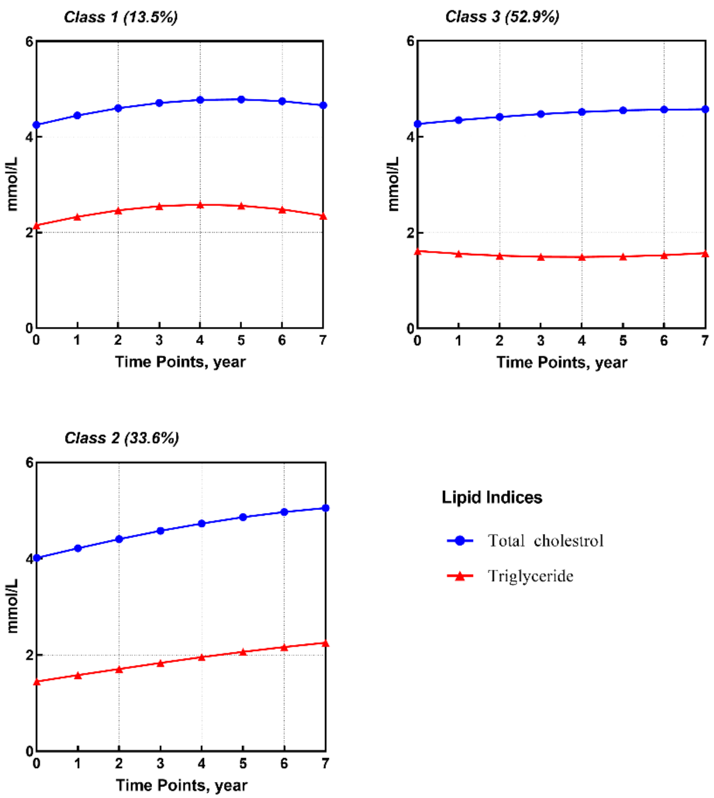

Supplemental Figure 4. FPG trajectories identified by GMM among patients without ART shift during follow-up (n =1296).

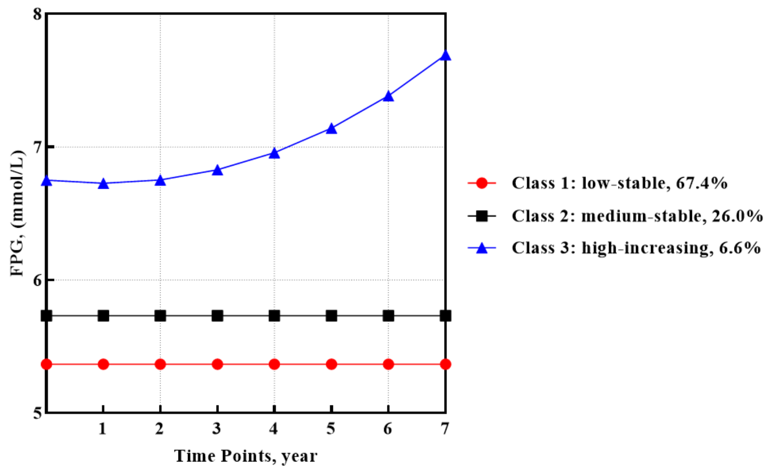

Supplemental Figure 5. Dual-trajectories of TG and TC identified by GMM among patients without ART shift during follow-up (n =1296).

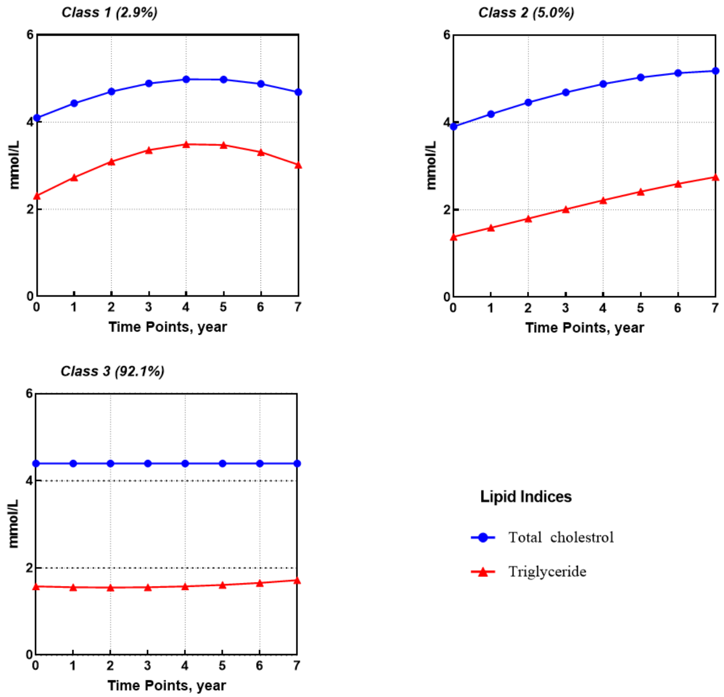

Supplement: Supplementary file 1 [file Data_Sheet_1.pdf]
